# melRNA-seq for Expression Analysis of SINE RNAs and Other Medium-Length Non-Coding RNAs

**DOI:** 10.1186/s13100-021-00245-z

**Published:** 2021-06-16

**Authors:** Yoshinobu Mori, Kenji Ichiyanagi

**Affiliations:** Laboratory of Genome and Epigenome Dynamics, Department of Animal Sciences, Graduate School of Bioagricultural Sciences, Nagoya University, Nagoya, 464-8601 Japan

## Abstract

**Background:**

Small interspersed elements (SINEs) are transcribed by RNA polymerase III (Pol III) to produce RNAs typically 100–500 nucleotides in length. Although their RNA abundance can be evaluated by Northern blotting and primer extension, the nature (sequence, exact length, and genomic origin) of these RNAs cannot be revealed by these methods. Moreover, mRNA sequencing (mRNA-seq) is not able to distinguish *bona fide* SINE RNAs or SINE sequences present in longer transcripts.

**Results:**

To elucidate the abundance, source loci, and sequence nature of SINE RNAs, we established a deep sequencing method, designated as melRNA-seq (medium-length RNA-seq), which can determine whole-length RNA sequences. Total RNA samples were treated with 5′ pyrophosphohydrolase (RppH), which allowed ligation of an RNA adaptor to the 5′ end of intact SINE RNAs. Similarly, another adaptor was ligated to the 3′ end, followed by reverse transcription, PCR amplification, size selection, and single-end deep sequencing. The analysis of two biological replicates of RNAs from mouse spermatogonia showed high reproducibility of SINE expression data both at family and locus levels.

**Conclusions:**

This new method can be used for quantification and detailed sequence analysis of medium-length non-coding RNAs, such as rRNA, snRNA, tRNAs, and SINE RNAs. Further, its dynamic range is much wider than Northern blotting and primer extension.

## Background

Many non-coding RNAs play various important roles in cell functions such as gene regulation, chromatin organization, RNA splicing and translation. Sequencing methods for regulatory small non-coding RNAs (typically, 18–35 nucleotides in length), namely miRNAs, siRNA, and piRNAs, have been well established based on ligation of an adaptor to both the 5′ and 3′ ends of RNA molecules. On the other hand, there is no established sequencing method for non-coding RNAs of medium length such as snRNAs and tRNAs.

Small interspersed elements (SINEs) are a class of retrotransposons widely distributed in eukaryotes that produce medium-length RNAs (typically 100–500 nucleotides) via RNA polymerase III (Pol III) transcription. SINEs proliferated by retrotransposition during evolution, and therefore a large number of divergent copies are present in each genome. The human Alu SINE and rodent B1 and B2 SINEs have been shown to play a role in regulation of gene expression. Although the abundance of SINE RNAs can be analyzed by Northern blotting and primer extension, the nature (sequence, exact length, and genomic origin) of these RNAs cannot be revealed by these methods. Regarding mRNA sequencing (mRNA-seq), many SINE copies reside in mRNA and long non-coding RNAs, which precludes distinguishing *bona fide* SINE RNAs from these longer transcripts. Small RNA-seq is not applicable either, since it only captures RNAs with 5′-monophosphate and 3′-hydroxyl ends, and SINE RNAs have a 5′-triphosphate (or capped) end which cannot ligate with an adaptor. In recent years, some methods for the sequencing and computational analysis of SINE transcripts have been developed [1–3]. These methods employed computational identification of SINE reads in polyA(+) RNA-seq data by excluding SINE reads in bodies of protein-coding and non-coding genes [1], utilize primer extension to selectively amplify SINE sequence of interest [2], and computational identification of reads from the Pol III transcription start sites in RAMPAGE data [3]. Although these methods are useful to delineate SINE expression in mammals, some limitations exist: the sequence information of exact 3′ ends are lost [1–3], the sequence information of SINE internal regions are lost [3], only polyA-containing SINEs [1] or primer-targeted SINEs [2] can be analyzed, and an internal control for comparison of SINE expression levels in different conditions is lacking [1–3].

**Fig. 1:**
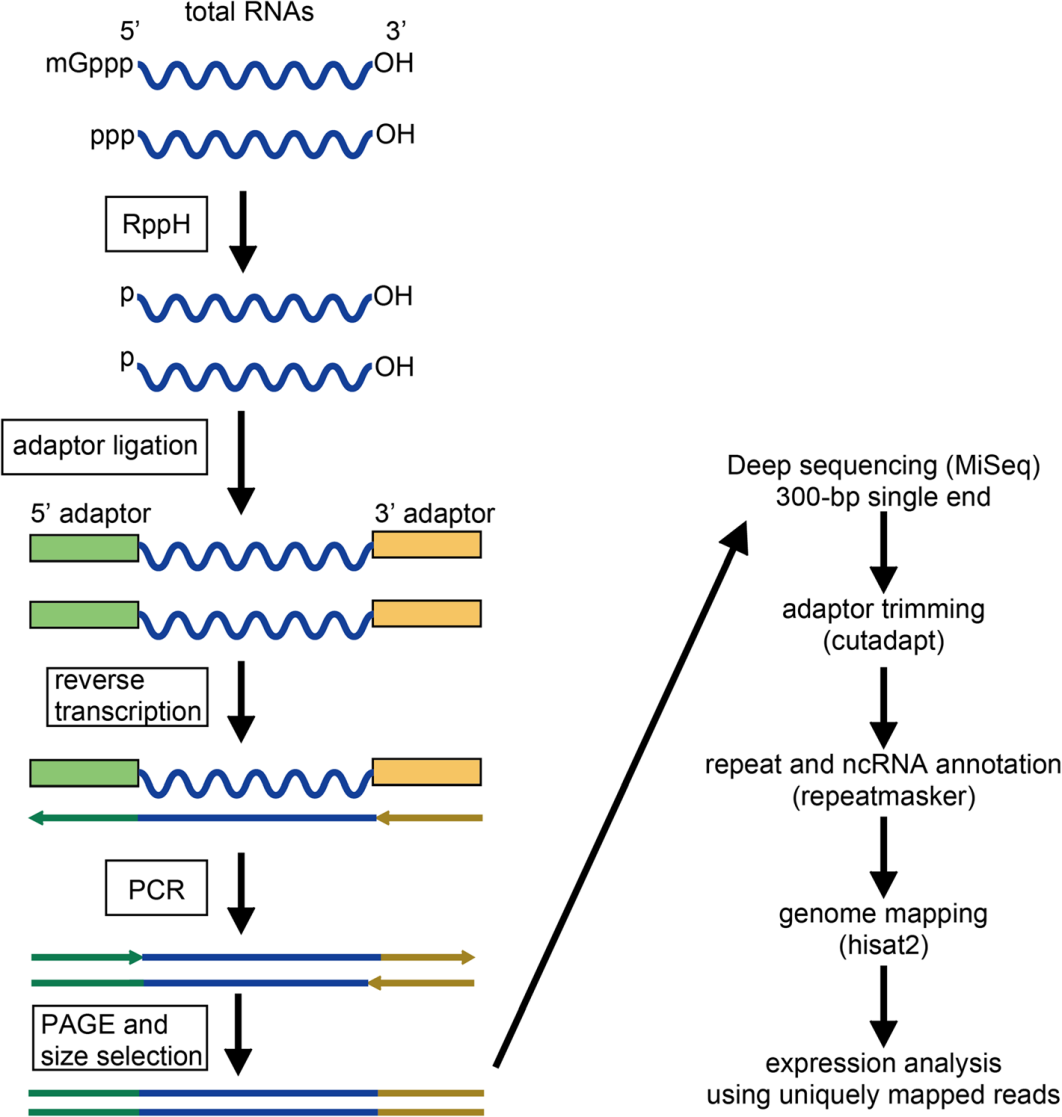
melRNA-seq analysis workflow. Schematic representation of melRNA-seq analysis. The experimental part consists of total RNA preparation, 5′ processing by RppH, adaptor ligation, reverse transcription, PCR amplification and size selection of the PCR products (left). After obtaining the sequences by MiSeq, they are trimmed to remove the adaptor sequence, and annotated by repeatmasker. Repeat-derived reads are mapped onto the reference genome (right). Mapped reads for individual loci are counted to calculate their expression levels

To elucidate the nature of SINE RNA sequences, we established a deep sequencing method, designated as melRNA-seq (medium-length RNA-seq), which can determine whole-length RNA sequences up to 600 nucleotides (Fig. 1). Herein, we describe how sequencing libraries are prepared and sequenced, and how obtained sequences can serve for the identification of SINE RNAs’ genomic origins. We also discuss its applicability to the study of other non-coding RNAs.

## Materials and Methods

### Mouse Tissues and Cells

Brain tissue was dissected from adult male mice (C57BL6/J). The spermatogonia were collected from testes at postnatal day 7 by fluorescence-activated cell sorting using an anti-EpCam antibody as described previously [4].

### Library Construction

Tissue and cell samples were dissolved in Isogen (Nippon gene), and total RNAs were prepared by Direct-zol RNA prep kit (Zymo Research). Although other RNA preparation methods are also applicable, we suggest avoiding RNA degradation to capture full-length RNAs. RNA (500 ng) was treated with 25 U of RNA 5′ pyrophosphohydrolase (RppH, New England Biolab) for 1 h at 37 °C in a 50-μl reaction mixture containing 1× Thermopol buffer (New England Biolab). This procedure freed 5′ cap or triphosphate into monophosphate, which can be later ligated by an RNA ligase. The reaction was stopped by phenol-chloroform extraction and ethanol precipitation. Sequencing libraries were constructed by using NEBNext Small RNA Library Prep kit (New England Biolab) according to manufacturer’s instructions. PCR products were run on a 6% native polyacrylamide gel, and a gel region corresponding to 240–380 bp (an insert size of 113–253 bp) was extracted. DNAs were eluted in 300 μl TE, ethanol precipitated, and dissolved in 20 μl of 0.1× TE. Library concentrations were determined by real-time RCR using the KAPA library quantification kit (KAPA Biosystems) on a StepOnePlus (Thermofisher).

### Sequence Analysis

The libraries were denatured in sodium hydroxide, neutralized in HT1 buffer (Illumina), diluted to 10 pM, mixed with the phiX174 control library (Illumina) (70% melRNA-seq libraries and 30% control), and sequenced on a MiSeq with MiSeq Reagent Kit v2 (Illumina) in the 300-bp single-end mode. About 6 million reads were yielded for each library. The sequences obtained by this method correspond to the sequences of the original RNA transcripts. Note that the libraries made by this method are also compatible with paired-end sequencing by MiSeq, HiSeq, and NovaSeq.

For sequence analysis, the adaptor sequence was removed from the obtained reads by cutadapt (https://cutadapt.readthedocs.io) with the option -a AGATCGGAAGAGCACACGTCT, and reads that did not contained the adaptor sequence were discarded. The retained reads were analyzed by repeatmasker (https://www.repeatmasker.org). Reads matched to the sense strand of a SINE sequence with “position in query start” and “position in repeat start” both ≤5 were retained. This procedure selected for RNA reads generated by transcription starting within 5 bp from the start nucleotide of the respective consensus sequences. The retained reads (about 78% of the reads containing a SINE sequence) were mapped onto the mouse reference genome (mm10) by hisat2 [5] with the option -a --score-min L,0,0 to retrieve all possible alignments without mismatch. About 95.5% of the reads were mapped uniquely, 2.8% were mapped 2 to 9 times, 0.8% were mapped 10 to 99 times, and the rest (0.9%) were mapped 100 or more times. The uniquely mapped reads were then used for counting the mapped reads for individual SINE loci by a Python script. The location and orientation of SINE loci were retrieved from the UCSC table browser [6]. For normalization of SINE expression, reads matching the sense strand of 5S or 5.8S rRNA (position in query start and position in repeat start both being 1 or 2, and query left and repeat left both being 5 or less) in the repeatmasker outputs were counted.

Because the repatmasker library does not contain the sequence of 5.8S rRNA, we added it to the library as below:

> 5.8S_rRNA#rRNA @Vertebrata_Metazoa GenBank: J01871.1

cgactcttagcggtggatcactcggctcgtgcgtcgatgaagaacgcagcgctagctgcgagaattaatgtgaattgcaggacacattgatcatcgacacttcgaacgcacttgcggccccgggttcctcccggggctacgcctgtctgagcgtcgct.

## Results and Discussion

### Sequencing Strategy and Validation

Figure 1 shows a schematic representation of the melRNA-seq method. To construct melRNA-seq libraries using total RNAs (in this study, RNAs in brain and spermatogonia), we first treated RNAs with 5′ pyrophosphohydrolase (RppH), which converted 5′ RNA ends with a cap or triphosphate into 5′ monophosphate ends. Then, unique adapters were ligated to the 5′ and 3′ ends of the RNAs, respectively by an RNA ligase using a commercially available small RNA-seq library preparation kit. After PCR amplification, the products were run on a 6% native polyacrylamide gel electrophoresis to select DNAs of approximately 240–380 bp in length (insert size of 113–253 bp). The library was sequenced by 300-bp single-end sequencing on MiSeq. After adaptor trimming, sequencing reads were analyzed by repeatmasker, which reports the position, orientation, and species of cellular RNAs (such as snRNAs and rRNAs) and transposable elements including SINEs.

**Fig. 2:**
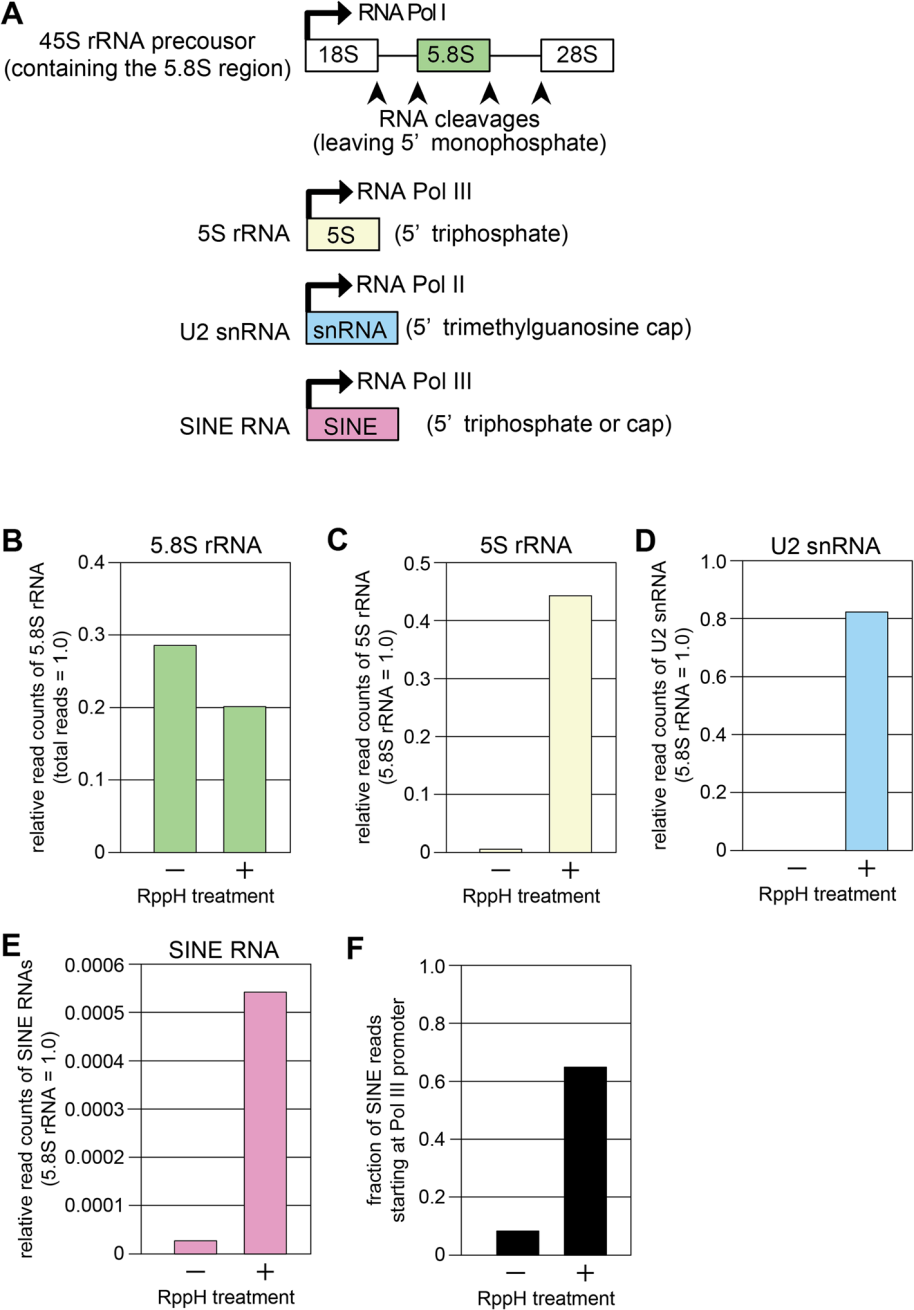
Validation of the methodological principle. **a** Schematic representation of transcription and post-transcriptional processing of 5.8S rRNA, 5S rRNA, and SINE RNA. **b** Fraction of 5.8S reads in total sequencing reads when brain RNA samples were treated (+) or not treated (−) with RppH. **c** 5S rRNA read counts relative to that of 5.8S rRNA when brain RNA sample were treated (+) or not treated (−) with RppH. **d** U2 snRNA read counts relative to that of 5.8S rRNA when brain RNA samples were treated (+) or not treated (−) with RppH. **e** SINE RNA read counts relative to that of 5.8S rRNA when brain RNA samples were treated (+) or not treated (−) with RppH. **f** Fraction of reads starting at Pol III transcription start sites in reads containing a SINE sequence when brain RNA samples were treated (+) or not treated (−) with RppH

As proof-of-principle, we checked the sequencing reads of two ribosomal RNAs (rRNAs) in the libraries prepared from brain RNAs. The 5S rRNA (121 nucleotides in length) is transcribed by Pol III and thus has a triphosphate at the 5′ end [7], whereas the 5.8S rRNA (158 nucleotides) is produced by enzymatic cleavage of the 45S rRNA precursor, thereby having a monophosphate at the 5′ end (Fig. 2a). Therefore, if total RNAs were not treated with RppH, only cleaved RNAs such as 5.8S rRNA could be ligated with the 5′ adaptor. Thus, the 5.8S rRNA reads were obtained regardless of the RppH treatment (Fig. 2b). Without the RppH treatment, the number of sequencing reads were > 200-fold higher for 5.8S rRNA than 5S rRNA (Fig. 2c, RppH(−)). When RNA was treated with RppH, the number of 5S rRNA reads increased by about 100-fold (Fig. 2c, RppH(+)). The U2 snRNA (188 nucleotides) is a Pol II transcript having a 5′ cap structure (trimethylguanosine) [7]. When RNA was treated with RppH, the number of U2 snRNA reads increased by about 400-fold (Fig. 2d). These results indicate that this method allows quantitation of intact RNAs with a 5′ cap or triphosphate. Likewise, the read count of SINE RNAs, transcribed by Pol III and having a triphosphate at the 5′ end, was 20-fold higher in the library with RppH treatment than in the library without (Fig. 2e). Moreover, the fraction of the SINE reads with correct Pol III transcription start sites was increased in the RppH(+) library (Fig. 2f). We have previously shown by Northern blotting that the expression level of SINEs in the brain is extremely low [4, 8]. Consistently, even with RppH treatment, SINE reads were much lower than 5S and 5.8S rRNA reads (> 30,000 times lower than 5.8S rRNA) in the brain RNA library.

### SINE Expression Analysis

**Fig. 3:**
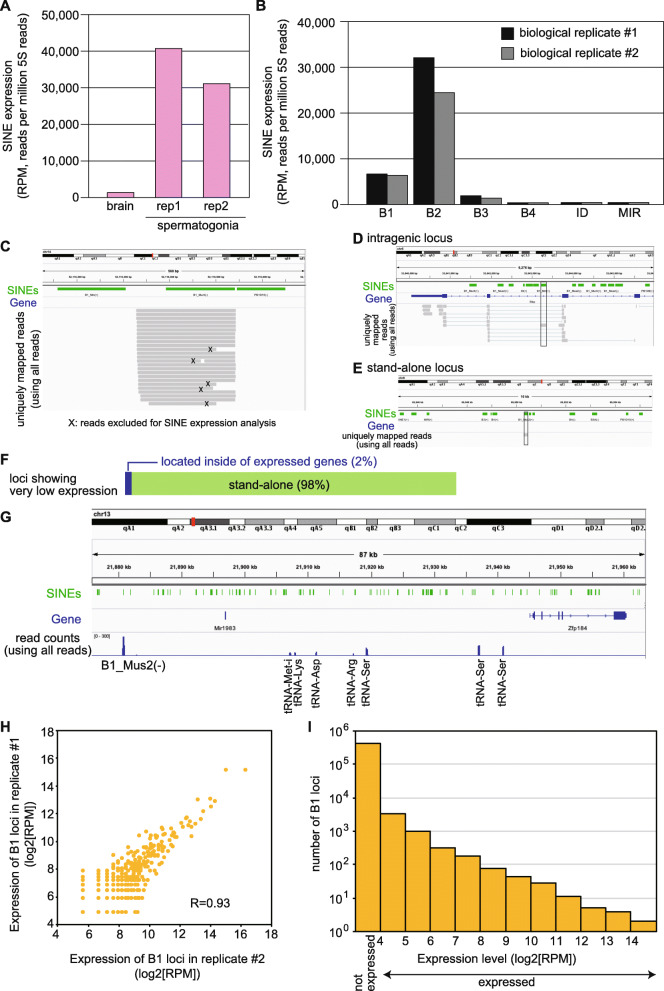
Reproducibility of melRNA-seq. **a** Normalized read counts (RPM, reads per million 5S RNA reads) of total SINE RNAs in the brain sample and two biological replicates (rep1 and rep2) of spermatogonium samples. **b** Normalized read counts (RPM) of B1, B2, B3, B4, ID, and MIR families in two biological replicates of spermatogonia samples. **c** An IGV snapshot showing all reads mapped around a B1_Mur4 locus (inserted in the minus-strand orientation). X indicates reads that were not transcribed from the Pol III transcription start site. **d** An IGV snapshot showing a poorly expressed B1_Mm locus that is inserted in an expressed gene in the same orientation. The reads with introns likely represent short RNAs produced by mRNA degradation. **e** An IGV snapshot showing a poorly expressed B1_Mus2 locus having no mapped read in its neighborhood (i.e., stand-alone locus). **f** Fraction of poorly expressed B1 loci (*n* = 1464) in terms of isolation of external transcription. **g** An IGV snapshot showing isolated expression of a B1_Mus2 copy inserted in the minus orientation to the genomic sequence. Read counts were calculated using all reads (i.e., regardless of the presence of a SINE sequence) that were uniquely mapped. Most of mapped reads outside SINEs in this region are mapped to tRNA genes. **e** Comparison of expression levels of individual B1 loci in the two spermatogonia samples. Pearson’s coefficient R (calculated using RPM values) is indicated. **d** Power-law distribution of the expression level of B1 loci in the spermatogonia. The histogram shows the loci number for the various expression levels indicated on the x axis. Expression levels correspond to the averages in the two replicates. The most left shows the number of loci where no read was mapped. The values of log2[RPM] ranges from 4 to 16 for expressed loci

To more rigorously validate whether the method is applicable for quantification of SINE expression, we prepared RNAs from spermatogonia, a type of male germ cells, because SINE expression in the testes was previously detected by Northern blotting [4, 8]. Total RNAs prepared from two individuals’ spermatogonia were respectively treated with RppH, and analyzed by melRNA-seq. Using the repeatmasker output files, we selected reads matching to the sense strand of SINE sequences with “position in query start” and “position in repeat start” both ≤5. These reads highly likely represnt *bona fide* SINE transcripts. Under these criteria, many reads (about 78% of reads containing a SINE sequence) were identified as SINE transcripts (Fig. 3a). To compare SINE expression levels between two spermatogonial libraries and a brain library, the number of 5S rRNA reads was used as internal control, as it reflects both the total read number and the efficiency of RppH-catalyzed conversion of 5′ ends. The level of SINE expression was about 30 times higher in the spermatogonia than in the brain (Fig. 3a). In spermatogonia, the B2 SINE family was the most highly expressed, B1 was marginally expressed, while other SINE families were much less expressed (Fig. 3b), consistent with the fact that only B1 and B2 are currently transpositionally active [8, 9].

To identify the genomic loci from which these SINE RNA reads were transcribed, reads were mapped to a mouse reference genome (Fig. 3c). About 96% were uniquely mapped, and used for the expression evaluation of individual SINE loci. It is formally possible that, for loci with a low number of mapped reads, these mapped reads represent digested products of longer RNAs rather than *bona fide* SINE transcripts. We thus examined whether other reads were mapped around these loci. A limited number of poorly expressed loci had mapped reads (using all reads, not selected by the presence of SINE sequences) around them likely due to the degradation of mRNAs of their host genes (Fig. 3d). However, vast majority had no mapped read in the same orientation in 5-kb regions around them (Fig. 3e), indicating that they are stand-alone in terms of transcription. These stand-alone loci accounted for 98% of the poorly expressed loci (Fig. 3f). These data strongly support that the mapped reads with a correct 5′ start site represent SINE RNAs transcribed by Pol III. Even for highly expressed loci, mapped reads were restricted to inside of the loci and absent outside (Fig. 3g). Expression of 3372 B1 loci (0.8% of genomic loci) was detected in spermatogonia in both or one individual(s). Importantly, the expression level of each locus was consistent between the two individuals (Fig. 3h), indicating a good reproducibility of the method. The expression level highly varied between loci up to three orders of magnitude, and showed a power-law distribution (Fig. 3i). B2 expression patterns were also similar to those of B1 (data not shown) and analyzed in detail in our recent report [8].

### 3′ RNA End Analysis

**Fig. 4:**
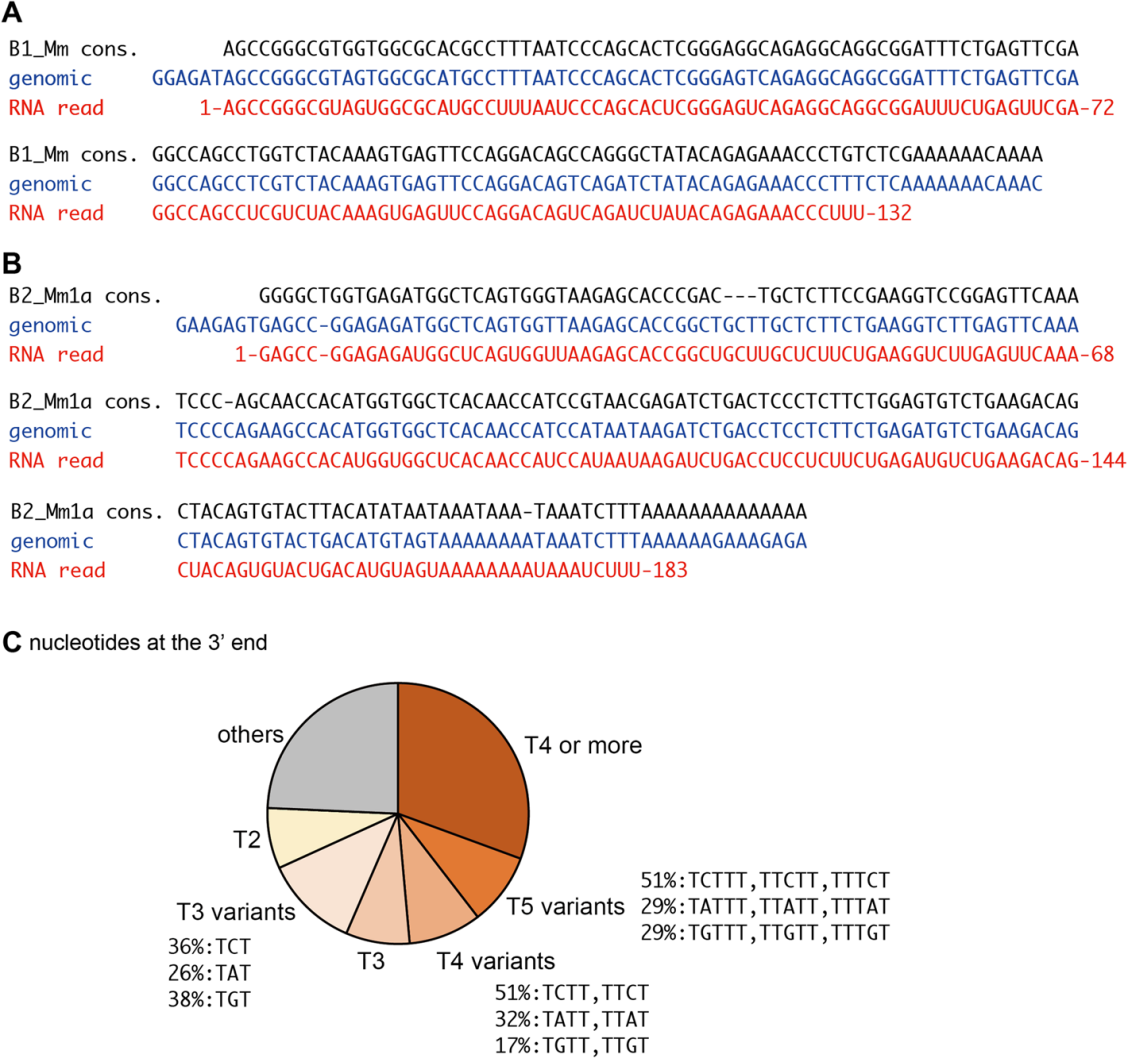
melRNA-seq identifies transcription termination signals. **a** An example of B1 RNA ending with 3 uridines (U3). The consensus sequence of B1_Mm (top, black), the genomic sequence (middle, blue), and the RNA sequence (bottom, red) are aligned. **b** Example of B2 RNA ending with U3. **c** Pie chart showing the statistics of nucleotide sequences in DNA templates around the 3′ end of SINE RNA (i.e., transcriptional termination signals). Fraction of subcategories for the variants are indicated. For classification, the order is T4, T5 variants, T4 variants, T3, T3 variants, T2, and other. For example, TTTCT is categorized in T5 variants (not T4 variants or T3)

Since whole RNA sequences are obtained by melRNA-seq, we were able to analyze the nucleotide sequence of 3′ RNA ends and the sequence of their DNA templates. It is known that Pol III transcription terminates in the DNA region with a series of thymidine in the non-template strand [10]. Indeed, 31% of SINE RNA reads ended at ≥4 consecutive thymines (T4). We also detected variants of T5 and T4, which have a single base substitution in T5 and T4, respectively, as well as T3, together corresponding to 26% of SINE RNA reads (Fig. 4). Although it is formally conceivable that these non-T4 SINE reads represent cleaved RNAs, its enrichment suggests that, in mice, a substantial portion of Pol III transcription terminates at sites that are deviated from ≥4 consecutive thymines. These results are consistent with the report by Oriori *et al.* [11] showing that similar variant sequences can trigger the termination of Pol III transcription *in vitro*.

### Comparison to Other Methods

Several methods have been reported for the large-scale analysis of SINE expression. Whereas they are useful for the study on SINE expression, there are some limitations. Conti et al. [1] utilized polyA(+) RNA-seq data and computationally excluded reads mapped to SINE loci inserted in bodies of protein-coding and non-coding genes in sense orientation. Thus, these loci are not able to be analyzed, and more importantly, the RNA-seq data lacks the sequence information of 5′ and 3′ ends due to random priming for library preparation. Although it readily applicable for published paired-end mRNA-seq data, we note that only SINE RNAs that have a polyadenosine stretch can be analyzed (Alu, B1 and B2 have it, whereas most other SINEs do not).

Kajijolich et al. [2] established a method called SINE-seq, in which SINE RNAs are reversed transcribed using a designed primer to make sequencing libraries. This allows the identification of 5′ RNA ends, but loses the 3′ end information. The method does not discriminate whether the 5′ end has a monophosphate (cleaved products), capped (Pol II transcripts), or triphosphates (others). Whereas a polyadenosine stretch is not prerequisite in this case, the method only captures RNA species that are complementary to the designed primer. Therefore, an internal control is absent unless another primer for a control is included in the reverse-transcription reaction.

Zhang et al. [3] established a method using RAMPAGE (RNA Annotation and Mapping of Promoters for the Analysis of Gene Expression) data. RAMPAGE [12], which has been used in the ENCODE project for gene expression analysis, can sequence the 5′ end of RNAs by the first read and the 3′ region by the second read in paired-end sequencing, which facilitates mapping of repetitive sequences. The sequence information at the 3′ end is lost due to the random priming step in the library construction. Depending on the sequencing length and RNA length, it also may lose the sequence information of the internal regions. In addition, to select *bona fide* SINE transcripts, it employs computational filtering using the entropy and length of mapped reads; therefore, the RNA detection is biased for highly expressed loci.

Advantages of melRNA-seq over the previous methods include the simple protocol for library preparation, the ability to determine the whole length of RNAs from the 5′ end to the 3′ end, no requirement for RNA sequence to be sequenced, selectable internal controls (such as 5S rRNA), and its applicability to studies on other non-coding RNAs as described below.

### Other Applications

We used a MiSeq 300-cycles kit for 300-bp single-end sequencing. Therefore, RNAs of ≥300 nucleotides could not be analyzed. However, 600-bp single-end sequencing (MiSeq) and 250-bp paired-end sequencing (HiSeq Rapid mode, NovaSeq) allow analysis of these RNAs, including human Alu SINE RNAs (although the length of Alu consensus sequences is about 290 bp, many Alu RNAs can exceed 300 nucleotides).

In the repeatmasker outputs, various snRNAs and tRNAs were also identified. Thus, the method offers an opportunity for the analysis of the expression of tRNAs, snRNAs, snoRNAs, etc. However, we note that additional steps may be required to ligate aminoacylated tRNAs and the adaptor.

## Conclusion

Herein, we present a new method for the quantification and sequence analysis of medium-length non-coding RNAs, such as rRNA, snRNA, and SINE RNAs. Like mRNA-seq and small RNA-seq, its dynamic range is much better than Northern blotting. Moreover, whereas quantification by Northern blotting depends on the degree of homology to the sequence(s) of the probe(s) used, melRNA-seq is independent. Moreover, source SINE loci could be identified by the method. Despite the source loci from “multiple hit” reads cannot be uniquely determined, repeatmasker allows identifying their subfamilies. As data produced by this method are highly reproducible, its applicability to many types of medium-length RNAs is guaranteed.

## Data Availability

The deep sequencing data has been deposited at NCBI GEO with the accession numbers, GSE171593 and GSE156315.

## References

[1] Conti A, Carnevali D, Bollati V, Fustinoni S, Pellegrini M, Dieci G (2015). Identification of RNA polymerase III-transcribed Alu loci by computational screening of RNA-Seq data. Nucleic Acids Res.

[2] Karijolich J, Zhao Y, Alla R, Glaunsinger B (2017). Genome-wide mapping of infection-induced SINE RNAs reveals a role in selective mRNA export. Nucleic Acids Res.

[3] Zhang XO, Gingeras TR, Weng Z (2019). Genome-wide analysis of polymerase III-transcribed Alu elements suggests cell-type-specific enhancer function. Genome Res.

[4] Ichiyanagi K, Li Y, Watanabe T, Ichiyanagi T, Fukuda K, Kitayama J (2011). Locus- and domain-dependent control of DNA methylation at mouse B1 retrotransposons during male germ cell development. Genome Res.

[5] Kim D, Paggi JM, Park C, Bennett C, Salzberg SL (2019). Graph-based genome alignment and genotyping with HISAT2 and HISAT-genotype. Nat Biotechnol.

[6] Navarro Gonzalez J, Zweig AS, Speir ML, Schmelter D, Rosenbloom KR, Raney BJ (2021). The UCSC Genome Browser database: 2021 update. Nucleic Acids Res.

[7] Busch H, Reddy R, Rothblum L, Choi YC (1982). SnRNAs, SnRNPs, and RNA processing. Annu Rev Biochem.

[8] Ichiyanagi T, Katoh H, Mori Y, Hirafuku K, Boyboy BA, Kawase M (2021). B2 SINE copies serve as a transposable boundary of DNA methylation and histone modifications in the mouse. Mol Biol Evol.

[9] Ichiyanagi K (2013). Epigenetic regulation of transcription and possible functions of mammalian short interspersed elements. SINEs Genes Genet Syst.

[10] Bogenhagen DF, Brown DD (1981). Nucleotide sequences in Xenopus 5S DNA required for transcription termination. Cell.

[11] Orioli A, Pascali C, Quartararo J, Diebel KW, Praz V, Romascano D (2011). Widespread occurrence of non-canonical transcription termination by human RNA polymerase III. Nucleic Acids Res.

[12] Batut P, Gingeras TR (2013). RAMPAGE: promoter activity profiling by paired-end sequencing of 5′-complete cDNAs. Curr Protoc Mol Biol.

